# Role of Chromatin Replication in Transcriptional Plasticity, Cell Differentiation and Disease

**DOI:** 10.3390/genes13061002

**Published:** 2022-06-02

**Authors:** Elena López-Jiménez, Cristina González-Aguilera

**Affiliations:** 1 Faculty of Medicine, National Heart and Lung Institute, Margaret Turner Warwick Centre for Fibrosing Lung Disease, Royal Brompton Campus, Imperial College London, London SW3 6LY, UK; e.lopez-jimenez@imperial.ac.uk; 2 Centro Andaluz de Biología Molecular y Medicina Regenerativa (CABIMER), Universidad de Sevilla-CSIC-Universidad Pablo de Olavide, 41092 Seville, Spain; 3 Departamento de Biología Celular, Facultad de Biología, Universidad de Sevilla, 41013 Seville, Spain

**Keywords:** chromatin replication, chromatin organization, cell identity, transcription regulation, parental histone recycling, epigenetic maintenance

## Abstract

Chromatin organization is essential to maintain a correct regulation of gene expression and establish cell identity. However, during cell division, the replication of the genetic material produces a global disorganization of chromatin structure. In this paper, we describe the new scientific breakthroughs that have revealed the nature of the post-replicative chromatin and the mechanisms that facilitate its restoration. Moreover, we highlight the implications of these chromatin alterations in gene expression control and their impact on key biological processes, such as cell differentiation, cell reprogramming or human diseases linked to cell proliferation, such as cancer.

## 1. Introduction

The accurate control of transcriptional programs is fundamental to sustain a correct cellular function and it requires a system that provides enough stability to maintain cell identity, but, at the same time, enough flexibility to respond to external stimulus. The studies from the last decades indicate that the cell ensures a correct gene expression regulation through different and complementary mechanisms. First, controlling the expression of specific transcription factors (TFs) that modulate the transcription of their target genes. Second, controlling the activity of these TFs by the way the genetic material is organized into chromatin [1,2]. In the eukaryote genome, 147 base pairs (bp) of DNA are wrapped around histone proteins to form the nucleosome, the basic unit of chromatin. Each nucleosome is formed by one tetramer of the histones H3 and H4 and two dimers of histones H2A and H2B. This association of nucleosomes with DNA contributes to maintain the stability of the genome and to regulate its accessibility to proteins [3,4]. Moreover, the presence of the epigenetic information in the form of post-translational modifications (PTMs) of both histones and DNA provides an extra layer of regulation modulating the recruitment of chromatin remodelers or TFs to the DNA [5,6]. Finally, the way the chromatin is spatially distributed within the nucleus creates the correct environment to induce a coordinated genome-wide regulation of gene expression [7,8,9].

Despite the relevance that the chromatin structure plays on cellular activity, in every cell division, there are two moments in the cell cycle where chromatin suffers a global disorganization: the S phase, where the DNA molecule has to be duplicated, and mitosis, where chromosomes are compacted and distributed into the two daughter cells [10,11]. Identifying the chromatin rearrangements that take place after these critical processes and knowing how the cells restore the genome organization is critical to understand key biological processes linked to cell division, such as cell differentiation, cell reprogramming or important human diseases, such as cancer.

Recent technological breakthroughs in high-throughput sequencing and microscopy have allowed the discovery of the nature of newly replicated chromatin paving the way to understand these fundamental questions in biology [12]. In the next sections, we highlight the latest scientific findings that have enabled us to better understand the chromatin changes produced after DNA replication and the mechanisms involved in its post-replicative restoration. Moreover, we discuss the impact of this chromatin alteration on gene expression regulation and its contribution to cell differentiation and human diseases. For details on chromatin rearrangements occurring during mitosis, excellent reviews can be found elsewhere [13,14,15].

## 2. Chromatin Replication

Chromatin replication starts during S phase, when thousands of replication forks fire coordinately in different part of the genome following a ‘replication timing program’ to copy the genetic material in a precise amount of time [10]. In each of these replication forks, a macromolecular complex called replisome copies the DNA molecule by the action of its two principal components, the replicative helicase and the DNA polymerases. The replicative helicase, or CMG complex, is formed by CDC45 (cell division control protein 45), the MCM2-7 complex (minichromosome maintenance complex 2–7) and GINS, and it is responsible for unwinding the two strands of the DNA ahead of the replication fork [16,17]. Behind the helicase, the newly replicated DNA is synthetized by the action of several DNA polymerases that work in a coordinated manner to copy both strands of the DNA helix. The initiation of DNA replication starts with the action of the DNA polymerase alpha (Polα). Polα is associated with the primase, an enzyme that synthetizes short RNA primers 7–12 ribonucleotides long, which is subsequently used for other DNA polymerases to elongate the DNA. Since the two DNA strands are intertwined with opposite polarity, one strand of the DNA is copied continuously (leading strand), while the other strand is synthetized in a discontinuous manner (lagging strand). The synthesis of the leading strand is performed by the action of the DNA polymerase epsilon (Polε). However, the lagging strand is synthetized by the repetitive action of Polα and the DNA polymerase delta (Polδ), forming the Okazaki fragments. Finally, the maturation of the Okazaki fragments requires the degradation of the RNA primers and the ligation of the DNA fragments by DNA ligase 1 [18,19].

**Figure 1:**
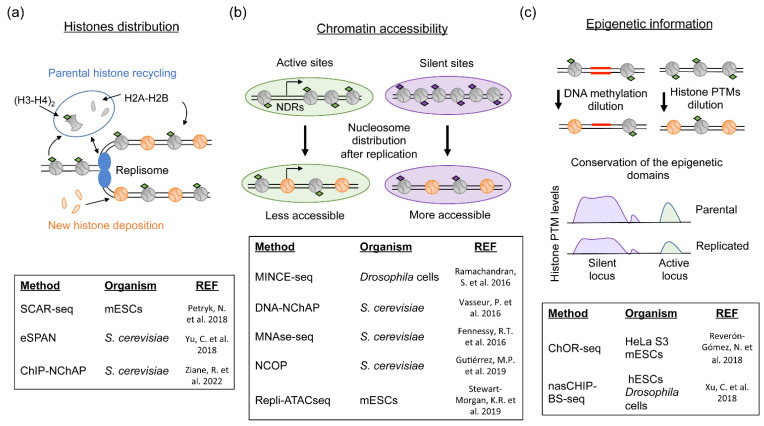
Chromatin replication and the chromatin changes observed in newly replicated chromatin during mitotic cell division. (**a**) Histone distribution. Nucleosomes are evicted ahead of the replication fork and segregated into H3–H4 tetramers and H2A–H2B dimers. Then, they are reassembled into the two newly synthetized DNA strands forming new nucleosomes. Parental histones are recycled conserving their PTMs and distributed symmetrically between the two sister chromatids. Some replisome components assist this replication-coupled process allowing the propagation of the epigenetic information. New histones have to be also incorporated to restore nucleosome density in the two daughter strands [29,30,31]. (**b**) Chromatin accessibility. After the pass of the replication fork, nucleosomes lose their pre-replicative positions and are transiently redistributed on nascent chromatin. On transcriptionally active sites, nucleosome-depleted regions associated with promoters are filled with nucleosomes and chromatin accessibility is reduced. In silent sites, chromatin accessibility increases, probably, due to a homogeneously and less compact nucleosome distribution [32,33,34,35,36]. (**c**) Maintenance of epigenetic information. DNA methylation levels are reduced after replication since newly synthetized DNA is not methylated. The incorporation of new histones that lack parental histone PTMs reduces by half the levels of pre-replicative histone marks. The recycling of parental histones close to their pre-replicative position allows the transmission of the epigenetic information maintaining the epigenetic domains. Tables in each panel indicate the genome-wide methods used to study parental histone recycling, chromatin accessibility and the maintenance of epigenetic information purifying newly replicated chromatin. Their references in the text are also included. NDRs: Nucleosome depleted regions; grey circles: nucleosomes containing parental H3–H4 tetramers; orange circles: nucleosomes containing new H3–H4 tetramers; black lines: DNA; red lines: DNA methylation; diamonds: Histone PTMs, arrow at NDR: Transcription start site and transcription directionality [37,38].

At the same time that the replisome ensures a faithful copy of the DNA molecule, DNA replication leads to a genome-wide chromatin disorganization. To allow the pass of the replisome, chromatin binding proteins, including TFs and RNA polymerases, should be detached from the DNA ahead of the fork to avoid blockages of the replication machinery and the generation of DNA damage [20]. This is also the case for parental nucleosomes that need to be evicted from DNA in a process that divides them into a H3–H4 tetramer and two H2A–H2B dimers [21]. To restore chromatin organization and maintain the stability of the DNA molecule after the pass of the replication fork, nucleosomes need to be reassembled into the two sister chromatids. Early studies of histone deposition on newly replicated chromatin have already shown that nucleosome assembly is a process coupled to DNA replication and that occurs in a stepwise manner where tetramers of H3–H4 are deposited first and the two dimers of H2A–H2B arrive later to form the nucleosome [21,22,23,24,25]. However, the presence of double amount of DNA requires not only the recycling of parental histones, but also the incorporation of newly synthetized ones to maintain nucleosome density in the two daughter strands (Figure 1a). The confirmation that parental histones were totally recycled came from a proteomic analysis conducted in human Hela S3 cells, which revealed that the same ratio of new and old histones was present in replicated chromatin [26]. Furthermore, it is also demonstrated that parental histones conserve their PTMs during the replicative process, providing an excellent way to propagate the epigenetic information [26,27,28].

The recycling of parental histones has been proven to occur in vivo, but a recent study using *Xenopus* egg extracts showed that parental histones were recycled with low frequency in vitro, indicating the need for active mechanisms to promote an efficient recycling of parental histones in vivo [39]. Histones are very basic proteins and need to be accompanied by the so-called histone chaperones until the moment they are incorporated into chromatin to avoid their aggregation and to prevent aberrant interaction with DNA or RNA [40]. The mechanisms of new histone assembly have been extensively studied and many histone chaperones have been associated with this process, including CAF-1 (chromatin assembly factor-1), ASF-1 (anti-silencing factor 1) and FACT (facilitates chromatin transcription) [41,42,43]. Meanwhile, the mechanism of parental histone recycling has remained elusive for a long time. Little is known about the mechanism for the recycling of H2A-H2B dimers, mainly due to their highly dynamic nature that makes difficult to analyze them [25]. Importantly, there is evidence that parental H3–H4 tetramers are segregated randomly between the two daughter strands for over three decades [44,45,46,47]. However, it was only during the last few years when two new genome-wide technologies, SCARseq and eSPAN, have started to shed light into the mechanisms governing the recycling of parental H3–H4 tetramers [48,49]. Combining the in vivo labelling of newly replicated chromatin with the purification and sequencing of DNA regions associated with histone PTMs and replicated areas, these techniques provided the first opportunity to study how PTMs associated with new and old histones are specifically distributed in each of the two replicated chromatids during mitotic cell division. These studies revealed that it is the proper replication machinery that promotes parental histone recycling and ensures the symmetric distribution of the parental H3–H4 tetramers in both nascent chromatids [29,30,50,51] (Figure 1a). The MCM2 subunit of the replicative helicase, together with the Ctf4 and Polα axis, were required to distribute parental histones in the lagging strand both in yeasts and mESC [29,50,51]. Conversely, the yeast Dpb3 and Dpb4 subunits of the DNA Polε were necessary for a correct parental histone recycling in the leading strand [30]. These results highlight the need for coordination between the replicative helicase and the DNA synthesis to achieve a correct parental and histone recycling. Indeed, treatments that block DNA synthesis producing the uncoupling between the replicative helicase and the DNA polymerases, such as hydroxyurea (HU), a ribonucleotide reductase inhibitor, have been shown to produce a defect of the local recycling of parental histones [52]. The role of these replisome factors in histone recycling is consistent with the histone chaperone activity described for MCM2 and Polε subunits and the presence of a histone-binding domains in their sequence [53,54,55,56]. However, a new study has questioned this model of parental histone recycling [31]. Using a genome-wide approach similar to that used in previous studies (ChIP-NChAP), they found that, in yeast, there was a difference of minutes in the replication timing of leading and lagging strands and that the old histones were preferentially assembled in the strand that replicates, first, independently of the MCM2 chaperone function [31]. In conclusion, despite the different models proposed, all these studies agree that the regulation of the parental histone segregation is closely linked to the replisome activity and replication process. However, additional studies are required to clarify the chaperone role of the replicative helicase and DNA polymerases in this process.

## 3. The Structure of Post-Replicative Chromatin

Chromatin accessibility to TFs and RNAPII depends on nucleosome occupancy and on nucleosome positioning. Nucleosome occupancy can be defined as the number of nucleosomes present in a specific location and nucleosome positioning indicates the place where the nucleosomes are located in a specific DNA sequence [57]. These events are tightly regulated and in transcriptionally active genes; promoters contain a nucleosome-depleted region (NDR) around the transcription start site. This is surrounded by two well positioned nucleosomes (called +1 and −1 nucleosomes) that are followed by regular spaced nucleosomes over the gene body [57]. Despite the recycling of parental histones and the restoration of nucleosome density after the pass of the replication fork, chromatin structure is altered, and the pre-replicative distribution of nucleosomes is transiently lost. The first evidence of post-replicative changes in nucleosome positioning came from a series of genome-wide studies performed in yeast and flies that used micrococcal nuclease (MNase) digestion to determine nucleosome distribution on S phase or newly replicated chromatin [32,33,34,35]. These studies revealed transient changes in the position of nucleosomes within the gene bodies of transcriptionally active genes and an increase in nucleosome occupancy in NDRs close to active promoters and enhancers immediately after the pass of the replication fork [32,33] (Figure 1b). Importantly, when comparing the distribution of nucleosomes containing active or silent histone marks, it was apparent that the post-replicative changes in nucleosome distribution was higher in active genes compared to low expressed ones or intergenic regions [35]. This nucleosome redistribution seems to be conserved in mammalian cells. In mESC, chromatin structure was studied by checking DNA accessibility in nascent chromatin with repli-ATACseq experiments [36]. Consistent with the results obtained in yeast and flies, a general decrease in DNA accessibility in the NDRs of promoters and enhancers regions was observed when newly replicated chromatin was purified. Later, these results were validated in the immortalized RPE-1 human cell line where chromatin accessibility data from synchronized S phase cells also revealed less accessibility in active regions. However, an increase in chromatin accessibility was observed in the silent regions associated with trimethylation on lysine 9 at histone 3 (H3K9me3), a mark linked to condensed heterochromatin [58]. Altogether, both the decrease in accessibility in active regions and the increase in silent regions would be consistent with a model in which after the pass of the replication fork, nucleosomes are homogeneously distributed on nascent chromatin, producing the loss of the pre-replicative nucleosome positions that will have to be reestablished with time (Figure 1b).

In addition to the changes in nucleosome distribution, the arrival of newly synthetized histones to nascent chromatin creates two new challenges to maintain chromatin organization and achieve epigenetic transmission. First, parental histones are enriched in H3 and H4 methylations, modifications that codified most of the epigenetics information. However, new histones lack these modifications and, instead, are largely unmodified, only containing acetylation on lysine 5 and 12 of H4 and helping in their nuclear transport and assembly into chromatin [26,59]. Therefore, the mixture of new and old histones on nascent chromatin produces a global two-fold dilution of the parental histone methylation pattern that needs to be restored to propagate epigenetic information to the daughter cells (Figure 1c). Interestingly, this dilution of the epigenetic information is not restricted to histone PTMs since DNA methylated regions are converted into hemi-methylated ones after the pass of the replication fork (Figure 1c). Second, new histones could displace parental histones from their original positions and potentially alter the epigenome distribution in the daughter cell. Despite the arrival of new histones and the alteration of nucleosome positioning observed after replication, the experimental results indicate that parental histones are recycled and located close to their original positions. This would facilitate the maintenance of specific histone PTMs in the same areas they were present before replication. This was already suggested by early studies and mathematical models based on yeast data that estimated that nucleosomes containing parental histones should be recycled in a radius of 400 bp from their original position [60,61]. However, the confirmation arrived recently, with the development of the ChORseq (Chromatin Occupancy after replication and sequencing). This novel technology is also based on the in vivo labelling of newly replicated chromatin combined with the purification and sequencing of DNA associated with proteins specifically on replicated DNA. However, contrary to SCARseq or eSPAN that measure the relative abundance of histone PTMs between chromatids, ChORseq was the first technique that allowed to study the distribution and abundance of histone PTMs and proteins on nascent chromatin [37,48]. ChORseq experiments in human cell lines revealed that both active and repressive histone methylation domains were conserved after the pass of the replication fork [37] (Figure 1c). Later, other similar methods validated these results in different model organisms [38]. This suggests that local parental histone recycling could recapitulate epigenetic information and facilitate the propagation of parental marks on new histones [37]. Indications of this local parental histone recycling have also been observed with biochemical analysis tracking tagged parental histones both in vitro, using *Xenopus* egg extracts, and in vivo in yeast [62,63]. However, a recent similar study tracking tagged parental histones in specific active and silent locus in mESC has questioned the importance of parental histone recycling for the epigenetic maintenance in active sites [64]. In this study, parental histones located in silent regions remained close to their replicative position, recapitulating what was observed in other organisms. However, in a subset of active loci, a replication dependent drop in the quantity of parental histone was observed arguing to non-local or less efficient histone recycling [64]. A similar decrease in nucleosome occupancy was also observed in active genes in yeast when comparing nascent and mature chromatin [35,63] and in HeLa by fluorescent imaging of parental H3 [52]. However, in these later cases, it was always associated with transcriptional activity. More studies are then needed to clarify whether there could be different mechanisms for parental histone recycling in active and silent sites.

## 4. Post-Replicative Chromatin Restoration

**Figure 2:**
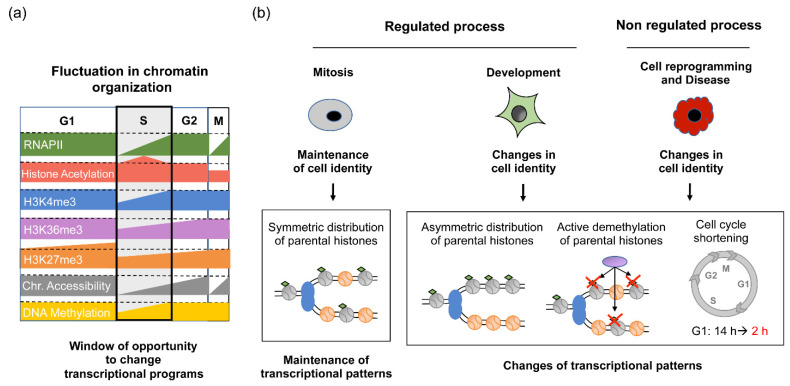
Impact of chromatin restoration on the maintenance of cell identity. (**a**) The different restoration kinetics of nucleosome positioning, chromatin accessibility and histone PTMs produce a fluctuation in chromatin organization and epigenetic information along the cell cycle. In mitotic cell division, pre-replicative chromatin organization is restored within one cell cycle. Chromatin accessibility, DNA methylation, histone marks associated with active sites as well as the levels of RNAPII are completely restored before cell division. However, histone marks associated with a repressed environment restore slowly and reach pre-replicative levels in the daughter cells. (**b**) The post-replicative alteration of chromatin structure could facilitate scheduled changes in transcriptional programs as occurs during development. However, if the chromatin changes are not controlled or the length of the cell cycle is altered, it could lead to unscheduled changes in cell identity linked to human diseases. We can also use the window of opportunity that creates chromatin replication as a tool to change artificially cell identity as happened during cell reprogramming. Grey and orange circles, black lines, and diamonds as in Figure 1; Purple circle: histone demethylase.

As shown above, studies from the last years leave no doubt that the replication of the genetic material alters chromatin organization. Now, the question that arises is how and how fast the cell can restore chromatin organization and whether the restoration process could be used as an opportunity to change transcriptional programs. During mitotic cell division, post-replicative chromatin alterations have been shown to be transient. Nucleosome positioning and chromatin accessibility are restored relatively fast in all regions of the genome and this fast restoration is conserved in all organisms. This is evident from the MNAse-seq and Repli-ATACseq studies mentioned above. In transcriptionally active sites, pre-replicative nucleosome position and chromatin accessibility are recovered in a matter of few hours after the pass of the replication fork [32,33,36] (Figure 2a). The same seems to hold true for human heterochromatic regions containing both the trimethylation of lysine 27 on histone 3 (H3K27me3) and trimethylation of lysine 9 on histone 3 (H3K9me3) [58]. Overall, these studies indicate that chromatin accessibility is restored before cell division in all parts of the genome except for some late replicating heterochromatin regions whose restoration should finalize in the next G1 phase.

Little is known about the mechanisms promoting the restoration of chromatin accessibility after replication. However, recent findings suggest that transcriptional activity could play an important role in this process. Indeed, the inhibition of transcription initiation results in a complete blockage of the restoration of chromatin accessibility in newly replicated active loci and the inhibition of transcription elongation delays chromatin accessibility restoration [36]. The precise mechanism by which transcription promotes this restoration is still unknown. Interestingly, a recent uncovered role of nascent RNA in the modulation of the binding to chromatin of chromatin remodelers could explain the need for active transcription to achieve an efficient chromatin restoration after replication [65]. However, transcription is not always required to restore chromatin accessibility since regions enriched on super enhancers or pioneer factor motives re-establish chromatin accessibility in a transcription-independent manner [35,36]. An interesting alternative mechanism has been proposed by a recent study that suggests that not only parental, but also new histone, PTMs could be playing an active role in post-replicative chromatin restoration. This is based on the observation that HAT1, the histone acetyltransferase responsible for the acetylation of new histones, is necessary to acquire a correct chromatin accessibility in heterochromatin domains in MEFs [66].

Despite the fast restoration of chromatin accessibility, the restoration of the epigenetic information occurs at different speeds depending on the mark. For example, the restoration of DNA methylation also occurs fast since the vast majority of pre-replicative methylated regions were completely methylated as soon as 20 min after the pass of the replication fork in embryonic stem cells [67] (Figure 2a). In the case of histone PTMs, a complete restoration of pre-replicative histone marks is achieved within one cell cycle, for all the histone PTMs tested. This ensures the transmission of the epigenetic information through cell division. However, restoration at active and silenced sites occurs at different speed. Histone PTMs levels associated with transcriptionally active loci restore faster than the ones associated with heterochromatin. The first evidence of the fast restoration of histone PTMs in active sites came from the measurement of human post-replicative levels of the trimethylation of lysine 4 on histone 3 (H3K4me3) by ChORseq analyses. These experiments revealed that less than one hour was enough to recover the pre-replicative H3K4me3 levels of highly transcribed genes, while less than six hours were necessary to complete restoration for lower expressed genes [37]. Then, ChIPseq and proteomic experiments studying the levels of other active marks, such as trimethylation of lysine 36 on histone 3 (H3K36me3), over different moments of the cell cycle also indicated that the restoration of this mark starts soon after the pass of the fork and is totally restored before cell division in the G2 phase [58,68]. On the contrary, in heterochromatic regions, much slower restoration dynamics have been observed. For example, in the case of H3K27me3, both in human and mESCs, proteomic, ChORseq and ChIPseq analysis indicated that the parental levels of this mark were completely restored in the next cell cycle immediately before the next round of replication [26,37,58,68] (Figure 2a). For H3K9me3, slow restoration kinetics have also been observed in humans, but not in mESCs [58,68], indicating that heterochromatin may not be completely established in mESC. Much of what we know about the mechanisms of histone PTM restoration is related to heterochromatic regions. Studies from the last decade have shown that the epigenetic restoration of heterochromatic histones PTMs (H3K27me3 and H3K9me3) follow a read and write mechanism where the pre-existing marks serve to recruit the enzymes that catalyze these marks and promote the further deposition of the mark in neighboring nucleosomes [69,70]. The slow kinetic of this process highlight the importance of cell cycle length to properly restore chromatin organization, especially to keep transcription repression. Moreover, considering the different restoration kinetics of the histone marks, a fluctuation in the epigenetic information is created along the cell cycle and could serve as a window of opportunity to change the epigenome and transcriptional programs (Figure 2a). Importantly, although elusive for long time, the mechanism responsible for the fast-epigenetic restoration at active sites is starting to be unveiled. A recent publication has demonstrated that the post-replicative restoration of the H3K4me3 levels in yeast depends on both the transcription machinery and the COMPASS complex being stimulated by the presence of the H3K4me3 mark itself [71]. Finally, in addition to transcription, the specific chromatin remodelers and the histone marks themselves, the moment when the DNA is replicated can also contribute to chromatin restoration after replication since an altered replication timing produces global alteration in chromatin modification and compartmentalization [72].

## 5. Consequences of Post-Replicative Chromatin Restoration in Gene Expression Control

Do post-replicative chromatin changes have any impact on gene expression control? To date, in mitotic cell division, there is no evidence of transcriptional activity in silenced genes after chromatin replication [58,73,74,75]. This argues that even half of histone PTMs levels that remain after replication should be enough to maintain gene silencing. This is consistent with the observation that the derepression of silent loci has only been observed after several rounds of cell division in conditions where no new histone methylation was allowed [51,68]. These results support the idea that the cell deposits an excess of histone PTMs in silent sites to ensure the correct repression of silent genes during replication and the maintenance of cell identity (Figure 2b).

In transcriptionally active sites, initiating RNAPII has been observed in nascent chromatin in less than 15 min after the pass of the replication fork that is long time before histone PTMs levels and chromatin accessibility are totally restored [31,36,37]. This could mean two things: that transcription could resume independently of the chromatin organization or that the reduced levels of the histones PTMs associated with transcribed regions are sufficient to promote transcription. Although the role of chromatin structure in the arrival of RNAPII and transcription restart is still under debate, now it is becoming clear that the post-replicative chromatin rearrangements do alter the transcriptional process both quantitatively and qualitatively. It has been shown that nascent chromatin is transcribed less efficiently. This is based on the observation that less RNAPII and less TFs are found in nascent chromatin [36,76,77]. Additionally, a recent analysis of nascent RNA in yeast and human cells revealed that transcription is also reduced immediately after replication [73,74,78]. This transcriptional reduction seems to be due to a reduction in the frequency of transcriptional bursts as shown by the single molecule imaging of post-replicative transcription in human cells [79]. Interestingly, it has been proposed that this transcriptional reduction may facilitate the buffering of gene expression required after doubling the DNA content during replication (Figure 2b). This would be consistent with the observation that no significant changes in global mRNA levels are observed before and after the pass of the replication fork in eukaryote cells [58,74,75,76], despite transcription in both sister chromatids has been observed [31,79]. Our current knowledge about the molecular mechanisms governing this buffering process comes mainly from yeast studies, which revealed that H3K56ac present in new histones and H3K4me levels are required for transcription buffering [76,78]. More analysis will be required to confirm whether similar chromatin mechanisms operate in mammals. Finally, in addition to the reduction in transcription levels, post-replicative chromatin alterations may also alter the regulation of where the transcription initiation events take place. This idea is based on the observation that novel accessible regions identified by Repli-ATACseq or MNAse-seq appear in gene bodies during the first minutes after the pass of the replication fork both in yeast and mESCs [35,36]. This would suggest that parental levels of histone PTMs may be required to avoid cryptic transcription initiation events.

## 6. Impact of Chromatin Replication in Development and Disease

During mitotic cell division, the cell needs to restore the pre-replicative chromatin organization to maintain cell identity and keep cellular function. However, there are specific biological events that require controlled changes in cell identity as occurring during cell differentiation in embryonic development or that are associated with unscheduled cell identity changes, such as in human cancer disease or during cell reprogramming. Usually, these cell identity changes are linked to an increase in cell proliferation. It has been proposed that post-replicative chromatin alterations may play a critical role in the regulation of these cell identity changes (Figure 2b). Epigenetic changes leading to alterations in transcriptional programs could be achieved by several means: the asymmetric distribution of epigenetic information, active remodeling of parental epigenetic information or modulating the cell cycle length. Examples of asymmetric histone distribution have been observed during asymmetric cell divisions that take place during development in which one cell maintains their pluripotency state and the other starts to differentiate. Using super resolution microscopy, the asymmetric distribution of new and old histones have been found in *Drosophila melanogaster* and mESC germline cells [80,81]. Interestingly, this asymmetric distribution of histones was correlated with unidirectional fork movement and the inheritance of old histone to leading strand [81]. An alternative mechanism to change cell identity is the active remodeling of parental epigenetic information. During the first hours of mammalian cell differentiation or during hematopoietic lineage specification, an active demethylation of H3K27me3 in nascent chromatin, mediated by UTX, was required for the binding of pioneer TFs [82,83]. The requirement for low levels of silencing marks during the early stages of differentiation is consistent with the observation that H3K36me2/3, associated with active transcription, and H3K27me3, associated with gene silencing, occupy mutually exclusive domains in the genome and that the alteration in the post-replicative restoration of the levels of one of these marks modified the levels of the other in the opposite direction [58,68]. Finally, a reduction in silencing marks can be also acquired by shortening the length of the cell cycle. Considering that the levels heterochromatic histone marks are restored very slowly after replication [26,37], the unscheduled acceleration of the cell cycle can compromise the maintenance of gene silencing in these regions. This could be particularly relevant during carcinogenesis where the overexpression of some of the most common oncogenes found in tumors, such as *MYC* or *CCNE1* (cycling E), shortened the length of G1 phase from 12–14 h to 2–4 h in U2OS cells [84]. Supporting this idea, conditions that blocked the post-replicative H3K9me3 restoration led to the activation of transposable elements (ERV1) after several rounds of cell division that could contribute to the genetic instability present in tumors [51]. Interestingly, these last two mechanisms could explain why cell reprogramming is more efficient when induction was performed in S phase synchronized cells [85] or when induction was performed selecting cells with a shorter G1 [86,87] by reducing the levels of repressive marks and facilitating the binding of Yamanaka factors to chromatin (Figure 2b).

## 7. Conclusions

In recent years, the development of new technologies linked to high-throughput sequencing and super resolution microscopy have allowed the direct study of newly replicated chromatin, revealing the nature of the chromatin rearrangements occurring after the pass of the replication fork. Nowadays, we know that, after chromatin replication in mitotic cell division, parental histones are recycled symmetrically into the two sister chromatids and placed close to their pre-replicative positions facilitating the transmission of the epigenetic information. However, the epigenetic information is diluted by half due to the incorporation of new histones and nucleosomes are redistributed altering chromatin accessibility. These changes are transient and are restored within one cell cycle, but we still know very little about the molecular mechanisms responsible for the restoration of chromatin organization and the maintenance of the epigenetic information. Importantly, we will have to face future challenges in the field, such as the elucidation of the role of replication machinery in the recycling of parental histones and the definition of the function of parental and new histone PTMs in the transmission of the epigenetic information. Moreover, it will be crucial to understand the impact of these chromatin changes in the regulation of transcription. Thus, the upcoming results in the next years will help to answer all these important questions and will pave the way to understand not only how cell identity is maintained during mitotic cell division, but also how these processes can contribute to key biological processes, such as cell differentiation, cell reprograming and tumorigenesis.

## Data Availability

Not applicable.
